# Effect of taxifolin on oxidative gastric injury induced by celiac artery ligation in rats^1^

**DOI:** 10.1590/s0102-865020190040000004

**Published:** 2019-05-06

**Authors:** Hüseyin Eken, Orhan Cimen, Ferda Keskin Cimen, Eray Kurnaz, Murat Yildirim, Volkan Tasova, Nezahat Kurt, Kamil Pehlivanoglu, Didem Onk, Asli Ozbek Bilgin

**Affiliations:** I Assistant Professor, Department of General Surgery, Faculty of Medicine, Erzincan Binali Yildirim University, Turkey. Scientific, intellectual, conception and design of the study; manuscript preparation.; II Assistant Professor, Department of General Surgery, Faculty of Medicine, Erzincan Binali Yildirim University, Turkey. Conception and design of the study, manuscript preparation.; III Assistant Professor, Department of Pathology, Faculty of Medicine, Erzincan Binali Yildirim University, Turkey. Histopathological examinations, manuscript writing.; IV MD, Department of General Surgery, Mengücek Gazi Training and Research Hospital, Erzincan, Turkey. Technical procedures, manuscript preparation.; V MD, Department of General Surgery, Zile State Hospital, Tokat, Turkey. Technical procedures, manuscript preparation.; VI MD, Department of General Surgery, Sabuncuoglu Serafettin Training and Research Hospital, Amasya University, Turkey. Technical procedures, manuscript preparation.; VII PhD, Department of Biochemistry, Faculty of Medicine, Ataturk University, Erzurum, Turkey. Acquisition, analysis and interpretation of data; technical procedures.; VIII Assistant Professor, Department of General Surgery, Faculty of Medicine, Erzincan Binali Yildirim University, Turkey. Manuscript preparation.; IX Assistant Professor, Department of Anesthesiology and Reanimation, Faculty of Medicine, Erzincan Binali Yildirim University, Turkey. Technical procedures, critical revision.; X Assistant Professor, Department of Pharmacology, Faculty of Medicine, Erzincan Binali Yildirim University, Turkey. Statistics analysis, manuscript writing, critical revision, final approval.

**Keywords:** Taxifolin, Celiac Artery, Ischemia, Reperfusion, Rats

## Abstract

**Purpose:**

To examine the effect of taxifolin on I/R induced gastric injury in rats using biochemical and histopatholohical methods.

**Methods:**

Eighteen albino Wistar male rats equally grouped as; gastric I/R (I/R), 50 mg/kg taxifolin + gastric I/R (TAX+ I/R) and sham operation applied (SHAM). Ischemia induced for 1 hour, and reperfusion induced for 3 hours.

**Results:**

Oxidant parameters like, Malondialdehyde (MDA) and Hydroxyguanine (8-OHdG) were higher, whereas total glutathione (tGSH) was lower in the I/R group according to SHAM group, histopathological findings such as marked destruction, edema, and proliferated dilated congested blood vessels were observed severely in the I/R group, whereas there was not any pathological finding except mild dilated congested blood vessels in the TAX+ I/R group.

**Conclusion:**

The taxifolin can be clinically beneficial in the treatment of gastric injury due to I/R procedure.

## Introduction

 Ischemia, as is known, is defined as oxygen deprivation in tissues as a result of the decrease or whole cessation of blood flow to living tissues. Reperfusion is the process of blood build up in ischemic tissue. However, reoxygenation in reperfusion results in xanthine oxidase, which is formed and accumulated during ischemia, to react with molecular oxygen and form excess free oxygen radicals^1^. Parks *et al.* ^2^ showed that reperfusion injury caused much more damage than ischemia alone due to this mechanism. Over-produced free oxygen radicals oxidize the lipids of cell membranes to produce toxic substances like malondialdehyde (MDA) from lipids. Also, free radicals react with DNA to cause oxidative damage in the DNA. One of the product of oxidative DNA damage is 8 hydroxyguanine (8-OHGua) and is used as an important parameter in determining oxidative stress^3^. In recent studies, the roles of increase in MDA production and decrease in total glutathione (tGSH) production have been shown in the pathogenesis of gastric ischemia-reperfusion (I/R) injury^4^. Polat *et al*.^5^ also reported that 8-OHGua was increased in parallel with the increase of gastric oxidative damage. Therefore, it was thought that drugs that inhibits the overproduction of MDA and excessive consumption of tGSH may be usefull in the treatment of gastric I/R.

 Gastrointestinal (GIS) mucosal tissue is the first region affected by the I/R event^6^. Gastric I/R damage can occur in shock, burns, trauma, vascular rupture, resection and other pathological conditions^6^ ^,^ ^7^. GIS I/R event leads to multiple organ failure which is the major reason of death in intensive care units^6^ ^,^ ^8^. This literature information indicates that antioxidant therapy may be beneficial in preventing or reducing GIS I/R injury. It’s reported in literature that endogenous antioxidant systems are insufficient for protection against oxidative stress induced by I/R^9^. For this reason, external antioxidant agents have been tested against GIS I/R damage and were found to be effective^10^. Taxifolin (3,3 ′, 4 ′, 5,7-pentahydroxiflavanon), which will be tested for its protective effects towards gastric I/R injury in the present study, has antioxidant properties. Also taxifolin is a flavanone found in milk thistle, onions, Douglas fir bark and French maritime^11^. And taxifolin shown to be metabolized in hepatosite cells and its presented that the major taxifolin metabolite is its sulfated conjugate, also the metabolites of methylation and reduction (dehydroxylation) were observed in studies^12^. In many studies, antioxidant activity of taxifolin has been shown^13^. Because of this feature, taxifolin prevented brain I/R damage^14^. These datas advise that taxifolin may be effective and useful in preventing gastric I/R injury. There are not found any studies in the literature investigating the effect of taxifolin on I/R induced gastric injury. Therefore, the purpose of this research is to investigate the effect of taxifolin on I/R induced gastric injury in rats using biochemical and histopathological methods.

## Methods

 Animal experiments were performed according to the National Guidelines for the Use and Care of Laboratory Animals and were confirmed by the local animal ethics committee of Atatürk University, Erzurum, Turkey (Ethics Committee Number: 75296309-050.01.04-E.1800138966, Dated:04.05.2018).

 Eighteen albino Wistar male rats of 256-270 grams weighing were used in the experiment. The rats were provided from Atatürk University Medical Experimental Application and Research Center. The animals were kept and fed in groups at normal room temperature (22°C) and under appropriate conditions before the experiment. 

### Chemicals

 Taxifolin used in the experiment was obtained from Evalar-Russia, and thiopental sodium was provided from I.E ULAGAY-Turkey.

### Animal groups

 Animals were divided into three groups: gastric ischemia reperfusion (I/R), 50 mg/kg taxifolin + gastric ischemia reperfusion (TAX+ I/R), and sham group (SHAM).

### Experimental procedures

#### Anesthesia

 All surgical methods on rats were applied under sterile conditions. Anesthesia was induced by 25 mg/kg intraperitoneal (i.p.) thiopental sodium and by making the rats sniff xyilazine at appropriate intervals. After thiopental sodium injection, rats were allowed to wait for the appropriate period of surgery. The time in which animals remain fixed in the supine position is regarded to be a appropriate period of anesthesia for surgery^15^.

#### Pharmacological and surgical procedure

 For the experiment, 50 mg/kg taxifolin was orally administered to the stomach by a probe. Using the same method, I/R and SHAM groups were treated with the same volume of distilled water as the solvent. 30 minutes after the application of taxifolin and distilled water, laparotomy was performed on rats under sterile conditions by a 2.5 cm long incision in the midline. For stimulating ischemia reperfusion damage, the celiac artery of TAX+ I/R and I/R groups was tied with a surgical thread and ischemia was induced for one hour. (No procedure was performed on the celiac artery of the SHAM group, and the abdominal region was closed with stitches). After one hour, ischemia was terminated, and reperfusion of the gastric tissue was induced for three hours^16^. At the end of the third hour of reperfusion, all group animals were sacrificed by high dose (50 mg/kg) thiopental anesthesia. Biochemical and histopathological examinations were performed on the gastric tissue taken from the sacrificed animals. Taxifolin was given to rats 30 min before ischemia/reperfusion procedure then ischemia was induced for 1 hour, and reperfusion was induced for 3 hours. And the gastric tissues was removed for biochemical and histopathological examinations from the rats nearly 4-5 hours after taxifolin administration. And the systemic effect of 50 mg/kg taxifolin against I/R injury was investigated^17^.

### Biochemical analyses

#### Malondialdehyde (MDA) analysis

 MDA analyses were based on the method used by Ohkawa et al., involving spectrophotometrical measurement of absorbance of the pink-colored complex formed by thiobarbituric acid (TBA) and MDA. The standard curve was obtained by using 1,1,3,3-tetramethoxypropane^18^.

#### Total Glutathione (tGSH) analysis

 According to the method defined by Sedlak J and Lindsay^19^ RH. DTNB (5,5’-dithiobis 2-nitrobenzoic acid) disulfite is chromogenic in the medium, and DTNB is reduced easily by sulfhydryl groups. The standard curve was obtained by using GSSG.

#### DNA oxidation analysis 

 The levels of 8-hydroxy-2 deoxyguanine (8-OHdG) and deoxyguanine (dG) were measured in pre-defined systems at various wavelengths by HPLC with HPLC-UV and HPLC-ECD electrochemical detectors. The dG and 8-OHdG amounts were identified using dG and 8-OHdG standards (Sigma, St. Louis, MO). 8-OHdG/10^5^ was given as DNA damage marker^20^. 

### Histopathologic examination

 Gastric tissues obtained from the rats were fixed in 10% formalin solution for 24 hours. After routine tissue processing, 4 micron thick sections were obtained from the paraffin blocks and were stained with Hematoxylin&Eosin. All sections were examined under the light microscope (Olympus BX 52, Tokyo, Japan) by two pathologists who do not know which treatment protocol is used. Severity of edema, dilated congested blood vessels and destruction assessed by following scoring; grade 0 represented no damage, grade 1 represented mild, grade 2 represented intermediate and grade 3 severe.

### Statistical analysis

 The results provided from the experiments are depicted as “mean ± standard error of mean” ( x ± SEM). The significance level of the inter-group difference was identified using one-way ANOVA test. Then, Tukey test was performed as post-hoc. For histopathologic comparisions Kruskal-Wallis test was used, as a post hoc Dunn’s test performed. All statistical analyses were performed using “IBM SPSS Statistics Version 20” program and p<0.05 was considered significant.

## Results

### Biochemical Findings

#### MDA, tGSH and 8-OHdG analysis results

**Figure 1:**
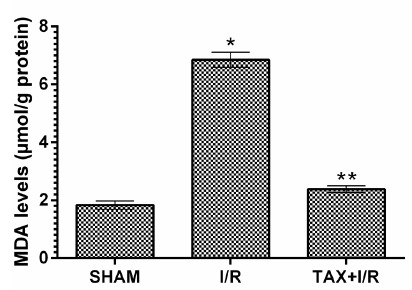
MDA levels in the gastric tissues of SHAM, I/R and TAX+I/R groups. (n=6), * = p <0.001, according to SHAM group, ** = p <0.001, according to I/R group.

 As can be seen in Figure 1, the MDA level in the gastric tissue of the I/R group was significantly higher compared to SHAM group (*p*< 0.001). Taxifolin administration decreased this elevation observed in the I/R group (*p*< 0.001). The difference in MDA level between TAX+I/R and SHAM group was not significant (*p*> 0.05) (Table 1). 

**Table 1:** Biochemical results of the gastric tissues of experimental groups.

Groups	Oxidant- Antioxidant parameters		
	MDA	tGSH	8-OHdG
SHAM	1.83±0.14	4.68±0.17	0.82±0.37
I/R	6.85±0,26*	1.87±0.67*	2.17±0.13*
TAX+I/R	2.38±0.12**	4.20±0.14**	0.99±0.63**

(n=6), * = *p* <0.001, according to SHAM group, ** = *p* <0.001, according to I/R group.

**Figure 2:**
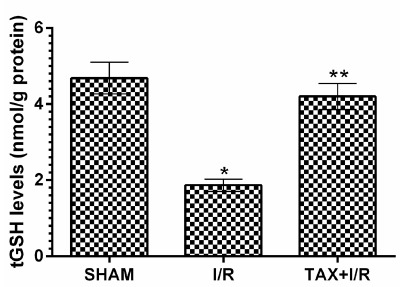
tGSH levels in the gastric tissues of SHAM, I/R and TAX+I/R groups. (n=6), * = p <0.001, according to SHAM group, ** = p <0.001, according to I/R group.

 In addition, Figure 2 shows I/R procedure caused a significant decrease in tGSH level in the gastric tissue compared to the SHAM group (*p* < 0.001). tGSH levels were higher in the taxifolin group according to I/R group (*p* < 0.001). The difference in tGSH levels between TAX+I/R and SHAM group was not significant (*p*> 0.05). 

**Figure 3:**
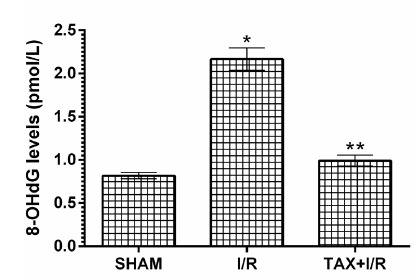
8-OHdG levels in the gastric tissues of SHAM, I/R and TAX+I/R groups. (n=6), * = p <0.001, according to SHAM group, ** = p <0.001, according to I/R group.

 In animals, I/R procedure increased the amount of DNA oxidation product 8-OHdG compared to SHAM group (*p* < 0.001). Taxifolin significantly prevented 8-OHdG elevation in gastric tissue due to I/R (*p* < 0.001).The difference between 8-OHdG levels in TAX+I/R and SHAM group was not significant (*p*> 0.05)(Fig. 3).

### Histopathological findings

**Figure 4:**
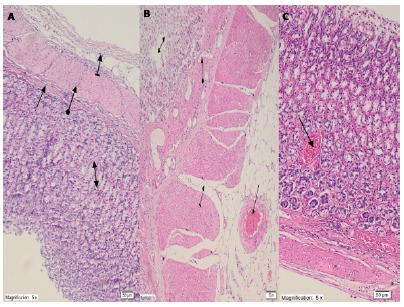
Histopathological appearance of the gastric tissues of rats in the study groups **A.** Gastric tissue of SHAM group: normal structures of muscularis mucosa (*straight arrow*), muscularis propria (*round arrow*), mucosa (*double arrow*) and serosa tissues (*striped arrow*) (HE x100). **B.** Animal group treated with ischemia-reperfusion in the stomach: destruction and edema of the gastric mucosa (*double-sided arrow*), partially proliferating dilated congested blood vessel (*round arrow*), edema (*striped arrow*) and dilated congested blood vessel in the serosa (*straight arrow*) (HE x200) **4C**. I/R-applied and taxifolin-treated gastric tissue: continuing dilated congested blood vessels in the mucosa (*straight arrow*) (HE x100).

Figure 4A shows the normal structures of muscularis mucosa, muscularis propria, mucosa and serosa tissues in the gastric tissue of SHAM group animals. There is no destruction (0), edema (0) and dilated congested blood vessels (0) in SHAM group, However, there is severe destruction (grade 3), edema (grade 3), and partially proliferated, dilated congested blood vessels (grade 3) in gastric tissue, and dilated congested blood vessels(grade 3) in the serosa of the I/R group in which ischemia-reperfusion was applied (Fig. 4B). There was not any pathological findings (edeme grade 0, destruction grade 0) in the gastric tissue of I/R animals treated with taxifolin except mild dilated congested blood vessels(grade 1) that only continued in the mucosa (Fig. 4C), comparisons between groups are presented in Table 2.

**Table 2:** Comparisons of histopathological findings of the gastric tissues of experimental groups.

Groups	Histopathological findings		
	Edema	Destruction	Dilated congested blood vessel
SHAM	0 (0-0)	0 (0-0)	0 (0-0)
I/R	3 (3-3) *p<0.001	3 (3-3) *p<0.001	3 (3-3) *p<0.001
TAX+I/R	0 (0-0) **p<0.001	0 (0-0) **p<0.001	1 (1-1) **p=0.41

Data are presented as the median (min-max).

*Compared to SHAM group, ** Compared to IR group, using Kruskal-Wallis and Dunn’s test.

## Discussion

 As is known, gastric I/R damage can occur in the resection process and other pathological conditions (shock, burns, trauma, vascular rupture), and cause multiple organ failure which is a reason of death^6^ ^-^ ^8^. Hence, research on the treatment of gastric injury and fatal complications due to I/R procedure is ongoing. In this experimental study, biochemical and histopathological effects of taxifolin on gastric I/R injury were investigated in rats. Biochemical parameters such as MDA, 8-OHdG and tGSH were used to evaluate the protective effect of taxifolin towards gastric I/R injury. It was also investigated whether the changes in MDA, tGSH and 8-OHdG levels were reflected in histopathological findings. Our results showed that I/R procedure significantly increased MDA level in gastric tissue compared to SHAM and taxifolin group. Our previous experimental study also presented that I/R procedure elevated MDA level in animal gastric tissue^4^. As it is known, MDA is last molecule of lipid peroxidation (LPO), also a good marker to evaluate oxidative tissue damage^21^. LPO is induced by free oxygen radicals (ROS). In liver tissues, ROS is produced by xanthine oxidase accumulated during the ischemic period reacting with the abundant molecular oxygen introduced to the tissue during reperfusion^1^. MDA was offered to be increased in I/R and different gastric injury models in many previous and recent studies^22^. This implies that our results are consistent with the literature.

 In this study, we also showed that I/R procedure increased 8-OHdG level in gastric tissue. In addition to MDA, 8-OHdG is also utilized to measure the severity of tissue I/R damage. ROSs also react with DNA and lead to oxidative damage of DNA^23^. Since DNA contains a large number of negatively charged phosphate groups, they are bound by positively charged metal ions such as Fe^+2/+3^ and Cu^+1/+2^ ^24^. These metal ions bound to the DNA react with the hydrogen peroxide (H_2_O_2_) present in the nucleus and cause the formation of toxic radicals such as •OH on the DNA and oxidative damage of the DNA^25^. 8-OHdGua is considered as the biomarker for oxidative DNA base damage^26^. DNA oxidation is known to have a significant role not only in I/R injury pathogenesis but also in carcinogenesis with various diseases^27^.

 This information suggests that suppression of ROSs and the reactions caused by ROSs will provide a treatment for the pathogenesis of I/R injury. As shown by our results, taxifolin significantly decreased MDA and 8-OHdG levels in the I/R administered gastric tissue. This indicates that taxifolin inhibit the reaction of ROSs on both membrane lipids and DNA. No previous studies indicating that taxifolin prevented gastric I/R injury were found, but it has been shown that I/R suppresses parameters that induce oxidative stress in brain tissue^28^. Information in the literature supports the view that excessive production of ROSs is responsible for I/R injury. Taxifolin was repoted to significantly antagonize the increase in ROS and MDA production in I/R-induced damaged brain tissue. Furthermore, it has been suggested that taxifolin shows antioxidant activity and protects brain tissue from I/R damage^14^. Experimental studies have proven that taxifolin inhibits the •OH radical in the cell and protects DNA from oxidative damage by showing antioxidant activity^29^. In this study, we found that taxifolin exhibited antioxidant activity by preventing the decrease in tGSH level in gastric tissue. Schlickmann *et al.* ^30^ reported that taxifolin antagonized the decrease in GSH level caused by oxidants in gastric tissue. GSH finds in many cells and is a tripeptide that consists of L-cysteine, L-glutamate and glycine. Catalyzed by the glutathione peroxidase enzyme which contains selenium in its active region, GSH reacts with H_2_O_2_ and organic peroxides and shows antioxidant activity by removing H_2_O_2_ from the cells. GSH detoxifies hydrogen peroxide or organic oxides chemically and protects cells from ROS damage^31^.

 Biochemical experimental results obtained in all groups showed that they overlapped with histopathological findings. Oxidant parameters such as MDA and 8-OHdG were higher in the I/R group, whereas tGSH was lower. Significant histopathological damage such as destruction, edema, and proliferated dilated congested blood vessels were found in the I/R group. However, destruction or edema was not observed in the taxifolin group, which showed MDA, 8-OHdG and tGSH levels similar to the SHAM group. Only congested blood vessels were seen in the taxifolin+ I/R group. It has been shown in the literature that ischemia increases the reverse diffusion of gastric acid and leads to LPO and tissue mucosal damage by ROS production after perfusion^32^.

## Conclusions

 The I/R process changed the oxidant antioxidant balance in the gastric tissue of the animals in favor of the oxidants.This indicates that the I/R process causes oxidative damage in gastric tissue of animals. Taxifolin changed the oxidant antioxidant balance to favor to oxidants in the I/R applied anima and suppressed the I/R related oxidative gastric damage . Also suppressing the elevation of oxidant parameters and reduction of antioxidant parameters, taxifolin significantly minimized the histopathologic damage induced by I/R in the gastric tissue. These findings suggest that taxifolin can be clinically beneficial in the treatment of gastric injury due to I/R procedure.
